# Expression of NOTCH3 exon 16 differentiates Diffuse Large B-cell Lymphoma into molecular subtypes and is associated with prognosis

**DOI:** 10.1038/s41598-018-36680-x

**Published:** 2019-01-23

**Authors:** Ditte Starberg Jespersen, Anna A. Schönherz, Hanne Due, Martin Bøgsted, Teis Esben Sondergaard, Karen Dybkær

**Affiliations:** 1 Department of Hematology, Aalborg University Hospital, Aalborg, Denmark; 2 Department of Chemistry and Bioscience, Aalborg University, Aalborg, Denmark; 3 Clinical Cancer Research Center, Aalborg University Hospital, Aalborg, Denmark; 4 Department of Clinical Medicine, Aalborg University, Aalborg, Denmark

## Abstract

Diffuse large B-cell lymphoma (DLBCL) is a heterogeneous disease with diverse clinical presentation and outcome. Bio-clinical prognostic models including oncogene expression and cell-of-origin phenotyping has been developed, however, approximately 30% of all patients still die from their disease, illustrating the need for additional prognostic biomarkers associating oncogenesis and phenotypic subclasses. Hence, we tested if alternative splice variations have biomarker potential. Initial alternative splicing analysis of human exon array from clinical DLBCL samples identified candidate genes. Experimental validation by ddPCR was performed in a DLBCL cohort classified into ABC/GCB subclasses, B-cell associated gene signatures (BAGS: naive, centroblast, centrocyte, memory, and plasmablast), and vincristine resistant gene signatures. Prognostic potential was assessed for aberrantly spliced transcripts. Thus, NOTCH3 was identified as alternatively spliced, with differential exon 16 depletion (−exon 16) between differentiation associated BAGS subtypes. Predicted vincristine resistant patients of the GCB subclass had significantly downregulated NOTCH3 −exon 16 transcript expression and tended to display adverse overall survival for R-CHOP treated patients. In conclusion, we have identified a specific alternatively spliced NOTCH3 event that differentiate molecular subtypes of DLBCL and display prognostic and predictive biomarker potential in GCB DLBCL.

## Introduction

Diffuse large B-cell lymphoma (DLBCL) is the most frequent lymphoid neoplasm among adults, accounting for 30% to 40% of all Non-Hodgkin’s lymphomas^1^. The immuno-chemotherapeutic regimen rituximab, cyclophosphamide, doxorubicin, vincristine, and prednisone (R-CHOP) is the standard treatment for primary diagnosed DLBCL, however, 30–40% of DLBCL patients eventually die from relapse or refractory disease^2–4^. DLBCL is a heterogeneous disease displaying diverse clinical presentation, outcome, cellular morphology, and pathogenic mechansism^1,5^. Hence, there is a need to identify and understand determinants of DLBCL heterogeneity to obtain better risk stratification of DLBCL patients^6^.

Based on gene expression profiling (GEP) DLBCL patients are classified into molecular subclasses with inherent prognostic impact: e.g. the ABC/GCB, the B-cell associated gene signature (BAGS) or the resistance gene signature (REGS) classification systems^7–9^.

Most widely used is the cell-of-origin classification system ABC/GCB consisting of activated B-cell (ABC) DLBCL, germinal center B-cell (GCB) DLBCL, and unclassified (UC) DLBCL, which recently have been included in the WHO guidelines^10^. The ABC/GCB subclasses differ in pathogenesis and survival outcome with ABC classified patients having an impaired 5-year survival rate compared to those classified as GCB when treated with R-CHOP^7^.

The BAGS classification is a refined cell-of-origin classification system where primary tumors at time of diagnosis are associated with normal B-cell subset phenotypes and classified into naive, centroblast, centrocyte, memory, and plasmablast B-cell subtypes providing additional prognostic information to molecular ABC/GCB subclasses. The most distinct prognostic impact of BAGS is observed within the GCB subclass, where the GCB-centroblast subtype display adverse survival outcome compared to the GCB-centrocyte subtype^8^. In addition, different activated signalling pathways, genetic profiles, and responses to chemotherapeutic drugs used in treatment of DLBCL is observed for the BAGS subtypes^8,9^.

The response of DLBCL patients to chemotherapeutic drugs of R-CHOP can be predicted by the REGS classification system, which is based on systematic *in vitro* dose response drug screens of B-cell cancer cell lines with cyclophosphamide, doxorubicin, or vincristine. Baseline GEP of untreated cell lines was combined with the degree of dose dependent growth inhibition after drug exposure. REGS allows assignment of a drug resistance probability to individual DLBCL patients, providing prognostic risk stratification of DLBCL patients^9^.

These molecular classifications have increased the biological understanding of DLBCL, however, it has recently been suggested that alternative splicing events and alternative exon usage have a significant role in the pathogenesis of DLBCL^6^.

The majority of human genes express alternatively spliced mRNA transcripts, contributing to proteomic diversity as well as tissue- and cell specific gene expression^11,12^. Alteration in the splice mechanism can contribute to malignant transformation, cancer progression, and metastasis^13–15^. Ratio modifications of normally occurring transcript variants, specific splice events, and cancer specific exon inclusion/exclusion are potential novel biomarkers and drug targets in cancer^16,17^. Previous studies have shown that expression of specific splicing events differs between prognostic subtypes of DLBCL and may hold biomarker potential^6,18–20^.

Increasing evidence has emerged that deregulated Notch signalling play a role in cancer progression. Mutations in NOTCH1 and NOTCH2 have been linked to haematological malignancies in several studies, including DLBCL^6,21–27^. Notch protein receptors (Notch1–4) differ structurally in their intra- and extracellular domain as they each participate specifically in conserved signalling pathways that regulate differentiation, cell cycle, proliferation, progression, and maintenance in development of several tissues and cell types, including lymphoid cells and self-renewal of hematopoietic stem cells^21,27–30^. In hematopoietic progenitor cells, Notch signalling promotes T-cell lineage commitment while inhibiting the B-cell lineage commitment^31^. Although, knowledge of the NOTCH genes involvement have increased in haematological malignancies, findings have recurrently been focused at the DNA level^26^. Since alternative splicing is a tightly regulated mechanism, altered splicing of NOTCH genes may affect B-cell lineage development and cause pathogenic transformations. Specific altered alternatively spliced events in NOTCH genes could be useful as molecular biomarkers in order to improve risk stratification of DLBCL patients.

**Figure 1:**
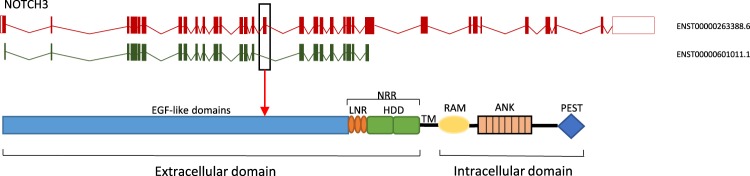
NOTCH3 transcripts and structural overview of the corresponding Notch3 protein. The upper panel shows the canonical NOTCH3 transcript (red) and the alternatively spliced transcript (green) missing exon 16 and 24–33. The lower panel presents the structure of the canonical Notch3 protein, which is a heterodimer consisting of a ligand-binding extracellular domain and an intracellular domain mediating target gene transcription. Exon 16 of the canonical transcript (box) codes for three EGF-like domains, which are absent in the alternatively spliced transcript. EGF: Epidermal growth factor, LNR: LIN12-NOTCH repeats, HDD: heterodimerization domains, NRR: negative regulatory region, TM: transmembrane domain, RAM: (RBPJ) association molecule, ANK: ankyrin repeats, PEST: proline (P), glutamine (E), serine (S), threonine (T) rich region.

In this study, we identified the alternative transcript of NOTCH3 missing exon 16 (−exon 16) (Fig. 1, ENST00000601011.1) by human exon expression profiles and subsequently experimentally validated the expression of alternative splicing events using droplet digital PCR (ddPCR) in a primary DLBCL cohort (n = 75). NOTCH3 excluding exon 16 was investigated for association with the DLBCL subclasses ABC/GCB, BAGS, and REGS vincristine subtypes documenting prognostic and predictive biomarker potential.

## Results

### Clinical characteristics

Baseline characteristics of the retrospective clinical cohort of 75 DLBCL patients are presented in Table 1. Mean age of the patients was 65 years (range 31–85). For identification of alternatively spliced genes, 37 primary DLBCL patients were analysed using HuEx. 1.0 array (Table 1). For subtype classification all 75 patients were analysed by HG-U133 Plus 2.0, including the 37 patients used in identification of candidate gene. For prognostic association, 60 R-CHOP treated patients with a median follow-up time of 4.6 years were analysed (Table 1).

**Table 1:** Patient characteristics of clinical DLBCL cohorts.

Characteristics	DLBCL HuEx. 1.0 cohort* n (%)	DLBCL cohort** n (%)	DLBCL R-CHOP treated cohort n (%)
	For identification of alternatively spliced genes	For subtype association	For prognostic assessment
*No. of patients*	37	75	60
***Gender***			
Female	17 (46)	32 (43)	23 (38)
Male	20 (54)	43 (57)	37 (62)
***Age, Years***			
Mean	63	65	65
Range	31–81	31–85	31–85
***IPI score***			
0–1	11 (30)	18 (24)	15 (25)
2–3	17 (46)	37 (49)	31 (52)
4–5	7 (19)	14 (19)	11 (18)
NA	2 (5)	6 (8)	3 (5)
***ABC/GCB***			
ABC	17 (46)	33 (44)	26 (43)
GCB	18 (48)	33 (44)	26 (43)
UC	2 (5)	9 (12)	8 (13)
***BAGS***			
Naive	3 (8)	9 (12)	5 (8)
Centroblast	8 (22)	13 (17)	11 (18)
Centrocyte	14 (38)	32 (43)	25 (42)
Memory	5 (13)	11 (15)	10 (17)
Plasmablast	7 (19)	10 (13)	9 (15)
***REGS vin***			
Sensitive	17 (46)	29 (39)	23 (38.3)
Intermediate	10 (27)	18 (24)	11 (18.3)
Resistant	10 (27)	28 (37)	26 (43.3)

IPI: International Prognostic Index, NA: not available, ABC: activated B-cells, GCB: germinal center B-cells, UC: unclassified, BAGS: B-cell associated gene signatures, REGS: resistance gene signatures, vin: vincristine. *Analyzed using HuEx. 1.0 array, **Subtype classification based on HG-U133 array.

### Identification of candidate genes using exon arrays

**Figure 2:**
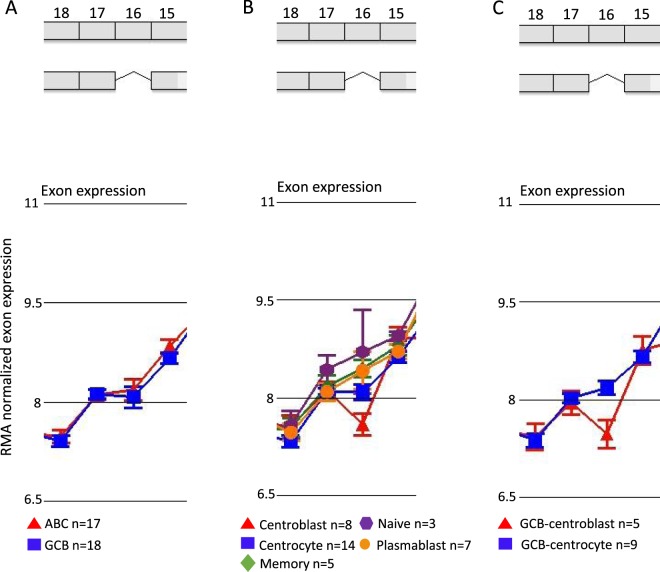
Exon specific expression of NOTCH3. RMA normalized exon specific expression assessed by HuEx. 1.0 array with focus on exon 15–18 of the NOTCH3 gene, as an alternatively spliced transcript missing exon 16 was identified, in DLBCL patients classified as (**A**) ABC/GCB subclasses, (**B**) BAGS subtypes, and (**C**) GCB-centroblast and GCB-centrocyte. Data points represent the mean probe set expression in the respective exons for each molecular subtype of DLBCL. ABC: activated B-cells, GCB: germinal center B-cells, CB: centroblast, CC: centrocyte, M: memory, and PB: plasmablast. RMA: Robust Multichip Average normalized.

Alternatively spliced genes associated with molecular subtypes of DLBCL were identified by the alternative splice analysis of variance (asANOVA) in 37 clinical DLBCL samples analysed by human exon arrays. Class comparison of ABC/GCB and BAGS classified patients listed 882 and 3442 candidate genes, respectively. Since the pronounced prognostic impact of the BAGS classification is present between the GCB-centroblast and GCB-centrocyte subtype^8^, class comparison was performed specifically for these two subtypes resulting in 193 candidate genes detected (Table 2). Selection of alternatively spliced candidate genes was based on an adjusted p < 0.05, visual inspection of exon expression across genes, and literature research. Thus, NOTCH3 emerged as the best candidate since exon 16 was alternatively spliced between the BAGS subtype centroblast vs. naive, plasmablast, memory, and centrocyte. Furthermore, exon 16 was alternatively spliced between the GCB-centroblast and GCB-centrocyte subtypes (Table 2). No difference in NOTCH3 exon expression was observed between ABC and GCB subclasses (Fig. 2A). Within the BAGS subtypes, however, centroblasts displayed lower exon 16 expression compared to the other subtypes (Fig. 2B), and when restricting the analysis to GCB-centroblast and GCB-centrocyte subtypes, loss of exon 16 was observed for the former (Fig. 2C), suggesting subtype specific exon 16 depletion in the centroblast subtype. Exon expression across the NOTCH3 gene are provided in Supplementary Figs 1–3.

**Table 2:** Identification of alternatively spliced candidate gene.

ABC/GCB (no. genes)	BAGS (no. genes)	GCB-CB/GCB-CC (no. genes)	Inclusion criteria
882	3442	193	asANOVA (FDR corrected p < 0.05)
50	50	50	Top 50
2 (FOXP1, **NLRP4**)	2 (**NOTCH3**, NID2)	4 (**NOTCH3**, LMO2, **NLRP4**, PRDM15)	Visual inspection of exon expression across genes (selected genes after visual inspection)^*^
FOXP1, NLRP4, NOTCH3, NID2, LMO2, PRDM15			Literature research
	NOTCH3		Selected alternatively spliced gene candidate

*Genes in bold are represented in more than one list. ABC: activated B-cells, GCB: germinal center B-cells, BAGS: B-cell associated gene signature, CB: centroblast, CC: centrocyte, asANOVA: alternative splice analysis of variance, FDR: False discovery rate.

To substantiate whether total gene expression of NOTCH3 could differentiate centroblasts from the other BAGS subtypes in DLBCL, all HuEx. 1.0 probe set expressions specific for each exons in NOTCH3 were extracted and summarized to a mean gene expression of NOTCH3, showing no significant difference between centroblast and centrocyte subtypes at gene level (Supplementary Fig. 4A). Thus, the NOTCH3 biomarker potential for distinguishing centroblast and centrocyte subtypes was observed only at exon level and not gene level. However, NOTCH3 gene expression differentiated the centroblast subtype from plasmablast subtypes (p = 0.027, Supplementary Fig. 4A). Similar when analysing healthy B-cells subset from tonsils association to NOTCH3 gene expression, no significance was found between the B-cell subsets (Supplementary Fig. 4B). However, when comparing DLBCL BAGS subtypes with healthy B-cell subsets a general lower expression of the NOTCH3 gene were observed in all of the healthy subsets from tonsils (Supplementary Fig. 4A,B).

### NOTCH3 −exon 16 transcript expression differentiates molecular subtypes of DLBCL by ddPCR

**Figure 3:**
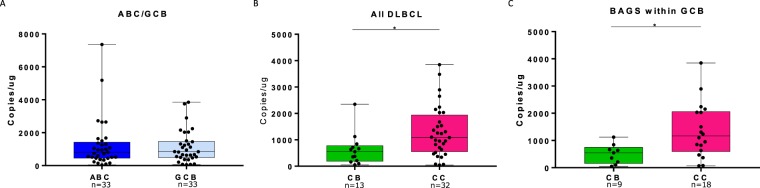
Association of NOTCH3 −exon 16 with molecular subtypes of DLBCL. Subtype association of NOTCH3 −exon 16 expression determined by ddPCR with (**A**) ABC and GBC classified DLBCL patients, (**B**) patients classified as centroblast and centrocytes, and (**C**) the centroblast and centrocyte subtype of GCB classified patients. ABC: activated B-cells, GCB: germinal center B-cells, CB: centroblast, CC: centrocyte. Median Whiskers boxplots were created with dots representing raw data points from maximum to minimum. *p < 0.05.

To analyse, if NOTCH3 −exon 16 was able to differentiate molecular subtypes of DLBCL, the expression were quantified in 75 clinical DLBCL samples using ddPCR and subtype associations were performed using unpaired t-test. Transcript expression of NOTCH3 −exon 16 did not reveal significant differences between ABC and GCB subclasses (Fig. 3A).

However, different expression patterns of the NOTCH3 −exon 16 transcript were observed between BAGS subtypes, displaying significant difference between centroblasts and centrocytes (p = 0.02, Fig. 3B), centroblasts and plasmablasts, and centrocytes and plasmablasts (p = 0.01, p = 0.04, respectively, Supplementary Fig. 5A). For BAGS subtype testing restricted to the GCB and ABC subclass, differential exon expression was observed within the GCB subclass alone (Fig. 3C, Supplementary Fig. 5B). In the GCB subclass, GCB-centroblast classified patients displayed lower NOTCH3 −exon 16 transcript expression compared to those classified as GCB-centrocyte (p = 0.02, Fig. 3C), an observation in accordance with exon array based results from Fig. 2C.

### Subtype specific exon usage of NOTCH3 exon 16 in molecular BAGS subtypes of DLBCL

**Figure 4:**
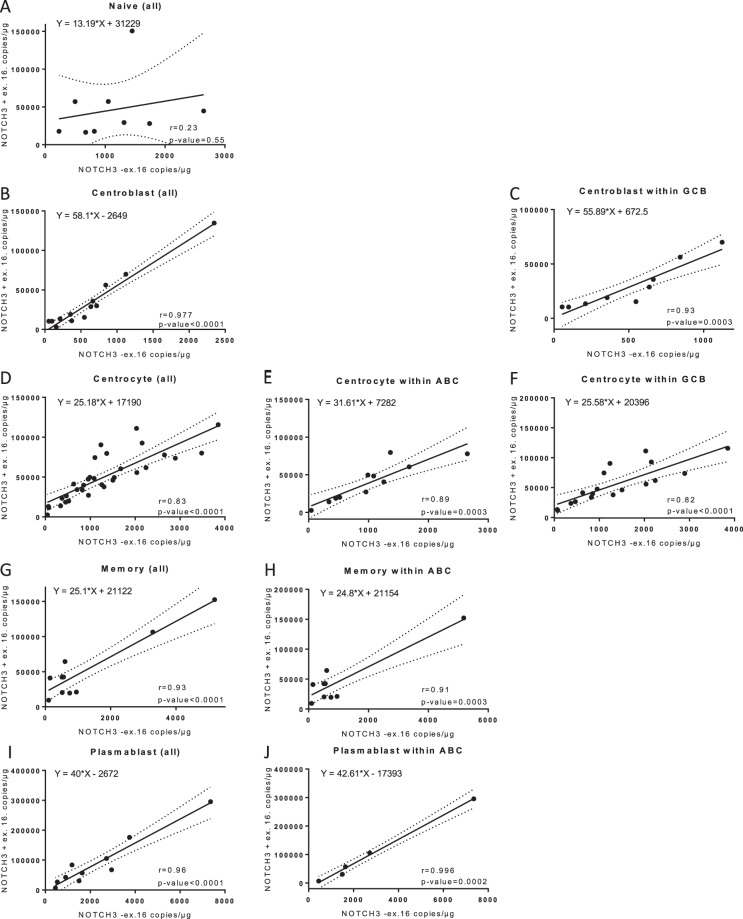
Correlation analysis of NOTCH3 +/−exon 16 expression in BAGS subtypes of all DLBCL patients and within ABC/GCB subclasses. Correlation of NOTCH3 +/−exon 16 in BAGS subtypes and BAGS within ABC/GCB subclasses, evaluated by Pearson’s correlation coefficient (r). Dashed lines represent the 95% confidence bands of the best-fit curve found by linear regression analyses. ABC: activated B-cells, GCB: germinal center B-cells, r: Pearson’s correlation coefficient.

Alternative exon expression of the NOTCH3 −exon 16 transcript were observed between BAGS subtypes, thus in order to investigate if the usage of NOTCH3 +/−exon 16 transcripts was subtype specific, ratios of NOTCH3 +/−exon 16 transcripts were assessed as the slope of the linear regression curve (Supplementary Table 1), while variation in expression was explained by correlation analyses (Fig. 4). In general, the NOTCH3 −exon 16 transcript was expressed at a much lower level than NOTCH3 +exon 16 as illustrated by slopes above 10 for all analyses (Supplementary Table 1). The naive subtype differed in slope and correlation coefficient (r) when compared to other BAGS subtypes, suggesting subtype specific exon usage and expression patterns (Fig. 4A, Supplementary Tables 1 and 2). In addition, the centrocyte and centroblast subtypes of the entire cohort and restricted to the GCB subclass had different NOTCH3 +/−exon 16 ratios (Supplementary Table 1, slope for centroblast: 58.1, centrocyte: 25.18, GCB-centroblast: 55.89, and GCB-centrocyte: 25.58), further supporting subtype specific expression (Fig. 4B–D,F). The centrocyte subtype had significantly lower correlation coefficient compared to the centroblast subtype, showing more variation in transcript expression within this specific subtype (p = 0.0046, Fig. 4B,D, Supplementary Table 2). For memory and plasmablast subtypes, high correlation coefficients and similar expression pattern of NOTCH3 +/−exon 16 were observed (Fig. 4G–J). Varying exon 16 usage in NOTCH3 and expression patterns between BAGS subtype, suggest that NOTCH3 exon 16 is expressed differently depending on which differentiation step the malignant B-cells are derived from.

### High expression of the NOTCH3 −exon 16 transcript is associated with vincristine sensitivity

**Figure 5:**
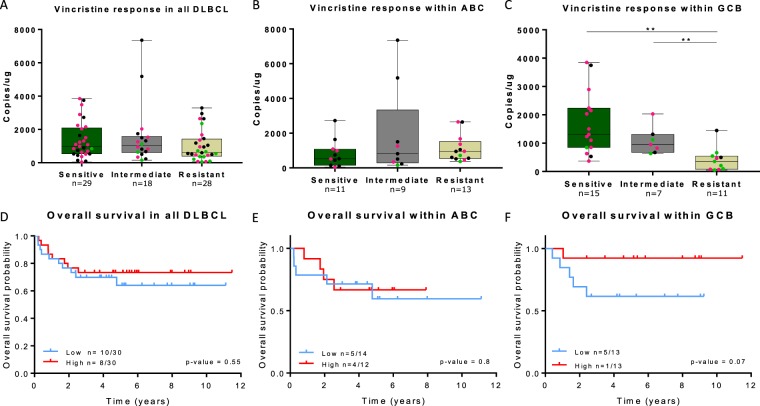
The NOTCH3 −exon 16 transcript associate with REGS vincristine subtypes and trends to associate with OS. Subtype association with NOTCH3 −exon 16 expression in vincristine response subtypes of (**A**) the entire cohort and (**B**,**C**) within ABC and GCB classified patients. Pink and green data points represent centrocyte and centroblast classified patients, respectively. (**D**) Kaplan-Meier estimates of NOTCH3 −exon 16 expression and OS in the entire DLBCL cohort (n = 60), (**E**) within the ABC subclass (n = 26), and (**F**) within the GCB subclass (n = 26). **p < 0.001. ABC: activated B-cells, GCB: germinal B-cells.

Each individual clinical sample was assigned a drug-specific response probability to vincristine, one of the cornerstones of R-CHOP, using the REGS classifier, dividing the entire cohort and ABC/GCB subclasses into three groups of vincristine sensitive, intermediate, and resistant (Fig. 5A–C). Association between the NOTCH3 −exon 16 transcript and vincristine response subtypes was observed within the GCB subclass (Fig. 5C). The NOTCH3 −exon 16 transcript was more highly expressed in DLBCL patients predicted to be vincristine sensitive compared to those classified as vincristine resistant (p = 0.001, Fig. 5C). The vincristine sensitive group of GCB classified patients mainly consisted of GCB-centrocytes, which previously has been predicted to be more sensitive to vincristine than GCB-centroblasts^8^. Additionally, the vincristine resistant group of GCB classified patients had significantly lower expression of the NOTCH3 −exon 16 transcript compared to the intermediate group (p = 0.006, Fig. 5C).

### Expression of NOTCH3 splice event trends to associate with prognosis in DLBCL

To evaluate the prognostic potential of NOTCH3 −exon 16 transcript expression, survival analyses were performed in R-CHOP treated DLBCL patients (n = 60) (Fig. 5D–F). Patients were separated into two equally large groups according to low and high expression of the NOTCH3 −exon 16 transcript. Differences between Kaplan-Meier estimates of survival probabilities for each group were determined using a log-rank test. Expression levels of NOTCH3 −exon 16 transcript were borderline associated with OS within the GCB subclass (Fig. 5F), whereas no prognostic stratification was obtained in the entire DLBCL cohort or in ABC classified patients (Fig. 5D,E). Further investigation of the NOTCH3 −exon 16 transcript as an independent prognostic biomarker was not pursued given the low sample size, yet the results suggests that expression of the NOTCH3 −exon 16 transcript has prognostic potential within GCB DLBCL.

### NOTCH3 −exon 16 transcript expression in healthy lymph node tissue

**Figure 6:**
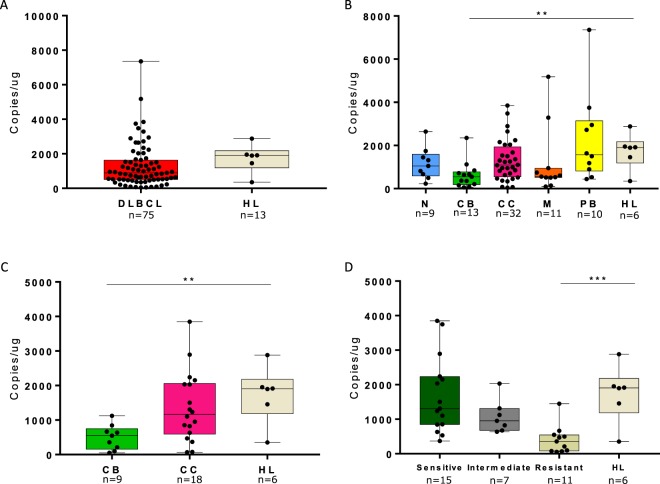
The NOTCH3 −exon 16 transcript expression in DLBCL compared to healthy lymph node controls. ddPCR quantified expression of the NOTCH3 −exon 16 transcript in healthy lymph node controls compared to (**A**) the entire DLBCL cohort, (**B**) BAGS subtypes, (**C**) BAGS subtypes within GCB, and (**D**) vincristine response in GCB classified patients. CB: centroblast, CC: centrocyte, HL: healthy lymph nodes, M: memory, N: naive, PB: plasmablast. **p < 0.001, ***p < 0.0001.

NOTCH3 −exon 16 transcript expression was able to differentiate DLBCL into molecular subtypes, however, when comparing the expression in healthy lymph nodes (n = 6) to the entire DLBCL cohort, the NOTCH3 −exon 16 transcript failed to discriminate DLBCL from healthy controls (Fig. 6A). In addition, expression of the centroblast, GCB-centroblast, and GCB vincristine resistant subtype of DLBCL was significantly downregulated compared to healthy lymph nodes (p = 0.004, p = 0.001, p = 0.0004, respectively, Fig. 6B–D), contradictory to the higher NOTCH3 gene expression observed in DLBCL BAGS subtypes compared to healthy B-cell subsets from tonsils at Supplementary Fig. 4A,B. However, the difference in NOTCH3 expression between healthy lymph nodes and the B-cell subsets was most likely affected by the different compositions of cells in the samples, since healthy lymph nodes consist of a mixture of cells, while the sorted healthy B-cell subsets consist of pure B-cells. Results indicate that the biomarker potential of the NOTCH3 −exon 16 transcript rely within stratification of DLBCL, and not as a diagnostic marker.

## Discussion

In this study, we identified NOTCH3 as an alternatively spliced gene and subsequently experimentally validated the biomarker potential in a primary DLBCL cohort. Patients were classified into molecular subtypes of DLBCL using GEP-based classifications, ABC/GCB and BAGS, which based on the B-cell degree of differentiation risk stratify DLBCL patients^7,8^ and REGS predicting vincristine response^9^. The alternative transcript of NOTCH3 missing exon 16 was selected as experimental candidate, since it displayed differential exon expression between BAGS subtypes and GCB-centroblast vs. GCB-centrocytes in the HuEx. 1.0 array analysis. Interestingly, when summarizing HuEx. 1.0 probe-expressions to a total NOTCH3 gene expression level, no differential expression was observed for ABC/GCB and most BAGS subtypes, contradictory to the result observed at exon level. Only the plasmablast showed significantly higher expression than centroblast at gene level. The loss of information at gene level is in agreement with Leivonen, *et al*.^6^, reporting that alternatively spliced events are able to discriminate the molecular ABC/GCB subclasses of DLBCL and have prognostic impact that is not seen at gene level.

Additionally, healthy B-cells subsets from tonsil tissue were analysed for the total NOTCH3 gene expression using HuEx. 1.0 arrays, showing no significant difference between healthy subsets. However, the expression of NOTCH3 for each healthy B-cell subset were higher compared to DLBCL subtypes. Especially, the DLBCL plasmablast had upregulated NOTCH3 gene expression compared to the healthy plasmablast subtype, which is in accordance with Delgado-Calle, *et al*.^32^ reporting that over-expression of NOTCH3 is observed in neoplastic plasma cells in Multiple Myeloma.

Despite a shift in detection platform, differential expression between BAGS subtypes and GCB-centroblast vs. GCB-centrocyte was confirmed using ddPCR, in which centroblasts displayed lower levels of the NOTCH3 −exon 16 transcript compared to centrocytes in accordance with exon array based findings. Differential expression of the NOTCH3 −exon 16 transcript in molecular subtypes was predominantly observed in the GCB subclass, implying that the NOTCH3 −exon 16 transcript expression is cell specific and may contribute to the heterogeneity of DLBCL. I addition, a significantly lower expression of the NOTCH3 −exon 16 transcript was observed in GCB patients classified as vincristine resistant by the REGS classifier, supporting the BAGS classification of DLBCL, which predicted centroblasts to be more vincristine resistant^8,9^. The role of altered alternative splicing in drug resistance of other malignancies have been demonstrated previously; cells expressing an alternative BRAF transcript lacking exon 4–8 showed increased resistance towards vemurafenib in melanomas^33^ and a truncated CD19 isoform without the segment encoded by exon 2 resulted in invisibility to CART-19 immunotherapy^34^. Implication of NOTCH3 in chemoresistance have been reported in breast and ovarian cancer^35–37^, however, to our knowledge, the role of the NOTCH3 −exon 16 transcript in vincristine response has not been described previously, yet current data support that NOTCH3 −exon 16 splice events have an effect on the vincristine resistance mechanism in DLBCL. However, to confirm link between alternative splicing of NOTCH3 and vincristine response, functional assays have to be performed.

The alternative splicing mechanism is tightly regulated in a tissue- and cell specific manner, where lymphoid tissue is documented to have one of the greatest enriched tissue-specific splicing^38,39^. Consequently, studies confirm that cell type dependent altered alternative splicing is implicated in the malignant pathogenesis, where Brown, *et al*.^19^ and Keimpema, *et al*.^20^ have documented that alternatively spliced transcripts of FOXP1 are overexpressed in the ABC subclass of DLBCL. Subtype specific ratios of NOTCH3 +/−exon 16 transcripts were observed for BAGS subtypes, suggesting specific splice regulation of NOTCH3 that could affect the function of NOTCH3 in different cell types depending on their degree of differentiation. The biological effect of exon 16 skipping and the cell specific NOTCH3 +/−exon 16 transcript interplay is unknown, yet we know that exon 16 encodes three EGF-like domains in the extracellular domain possibly affecting the protein-ligand interaction in a cell specific manner.

To evaluate the prognostic biomarker potential of the NOTCH3 −exon 16 transcript, R-CHOP treated patients were divided into two groups of high and low expression level and analysed for association with clinical outcome. NOTCH3 −exon 16 transcript expression exhibited prognostic potential in GCB classified patients, indicating that GCB patients with low expression display inferior OS. Although, clinical outcome is a result of the entire R-CHOP regimen, GCB classified patients with low expression levels of the NOTCH3 −exon 16 transcript was predicted to be vincristine resistant, which could potentially affect the survival outcome since vincristine is a cornerstone for efficacy of the R-CHOP regimen. Notably, no association with OS was observed when investigating the overall cohort or when restricting to ABC classified patients, supporting that altered NOTCH3 −exon 16 transcript expression has a different impact depending on molecular subtypes of DLBCL. However, only a trend was observed within the GCB subclass, most likely due to the relative small sample size of our cohort. Therefore, more studies using a larger independent patient cohort are required to confirm the impact of the NOTCH3 −exon 16 transcript on OS of GCB DLBCL patients.

In conclusion, we can experimentally confirm that quantification of alternative splicing events at exon level can be used to discriminate molecular subtypes of DLBCL. Evidence support that deregulated NOTCH signalling is a pathogenic driver in different haematological cancers^23–25,27^, however, most studies focus on NOTCH1 and NOTCH2 at DNA level. Here we document that the alternative splicing event in NOTCH3 mRNA show potential as biomarker for differentiating molecular subtypes of DLBCL. Specifically, when restricted to the GCB classified patients, NOTCH3 −exon 16 transcript expression demonstrates potential as a prognostic and predictive biomarker since patients with low expression of NOTCH3 −exon 16 transcript tend to have an adverse OS and be more resistant towards vincristine. This pilot study indicates that altered alternative splicing contributes to the pathogenesis of DLBCL depending on the molecular subtypes and may be promising as prognostic and predictive biomarker.

## Materials and Methods

### Patients

A retrospective cohort of optimal cutting temperature (OCT) compound embedded tissue biopsies collected at Aalborg University Hospital consisted of 75 *de novo* DLBCL patients. Tumor samples from DLBCL patients were collected at time of diagnosis in agreement with the RetroGen research protocol, reviewed and approved by the health ethic committee of the North Denmark Region (Approval jr. no. N-2014009) allowing exemption from the requirement of informed consent according to Sections 3 to 5 in Danish Act on Research Ethics Review of Health Research Projects. Informed consent was waived since this notifiable database research project did not involve any health risks and under the given conditions could not otherwise put a strain on the trial subject. Additionally, it would be impossible or disproportionately difficult to obtain informed consent or proxy consent, respectively, due to archival samples going back as far as 1990 and that several patients have died since collection. Diagnostic assessment of biopsies were performed by experienced hematopathologists. Of the 75 patients, 60 patients were treated in accordance to the standard protocols of R-CHOP-like regimens (R-CHOP cohort) and none were treated with stem cell transplantation. Patient characteristics are summarized in Table 1. Lymph nodes from healthy donors (n = 6) were collected in accordance with the research protocol (MSCNET, N-20080062MCH) accepted by the local ethic committee of North Denmark Region. Mononuclear cells from healthy tonsils (n = 6) were sorted using multiparametric fluorescence-activated cell sorting (FACS) with 8 different surface markers obtaining B-cell subsets of naive, centroblast, centrocyte, memory, and plasma blast as previously described^40^.

### RNA isolation

Total RNA was extracted using a combined protocol of TRIzol reagent (Invitrogen, Paisley, UK) and mirVana miRNA Isolation Kit (Ambion/ThermoFisher Scientific, Grand Island, NY) as previously described^8^. RNA concentration and integrity was determined using the Nanodrop ND-1000 spectrophotometer (ThermoFisher Scientific, Wilmington, DE, USA) and the Agilent 2100 Bioanalyzer (Agilent Technologies, Santa Clara, CA), respectively.

### Gene and exon expression profiling

Gene expression profiling of DLBCL patients (n = 75) was performed using Affymetrix GeneChip HG-U133 Plus 2.0 arrays (Affymetrix, Santa Clara, CA) according to manufacturer’s instructions. In parallel, RNA from 37 of the DLBCL patients and 30 healthy sorted B-cell subsets from tonsil tissue were labelled and hybridized to Affymetrix GeneChip HuEx. 1.0 ST arrays (Affymetrix), as described by the manufacturer. CEL files were deposited at the NCBI GEO repository (HG-U133 Plus 2.0: GSE110376, HuEx 1.0 ST: GSE110548) and comply with MIAME requirements.

### Droplet digital PCR

First-stranded cDNA was synthesized from 500 ng total RNA using SuperScript III First-Strand Synthesis SuperMix assay (Invitrogen) according to manufacturer’s instruction. Transcript expression of NOTCH3 with or without (+/−) exon 16 were quantified in DLBCL patients (n = 75) and healthy lymph node controls (n = 6) using QX200 ddPCR (Bio-Rad, Hercules, CA, USA). In brief, 5 µL of 12.5 ng/µL RNA-equivalent cDNA and 1 µL 900 nM primers/250 nM probe of each NOTCH3 +exon 16 (Hs00166432_m1) and NOTCH3 −exon 16 (APMFWV3) TaqMan assays (ThermoFisher Scientific) were mix with 11 µL of ddPCR Supermix for Probes (2x) (Bio-Rad). Nuclease-free water was added giving a total reaction mix volume of 22 µL. The +exon 16 assay amplifying NOTCH3 exon 16–17 has a VIC reporter, while the −exon 16 assay amplifying the 15–17 exon junction has a FAM reporter. Droplets were generated using Droplet Generation Oil for Probes (Bio-Rad) and 20 µL reaction mix, transferred to a 96-well PCR plate, and an endpoint PCR run was performed using GeneAmp PCR System 9700 (Applied Biosystems, Foster City, CA, USA) with the following program: 95 °C for 5 minutes, 40 cycles of 94 °C for 30 seconds, and 60 °C for 1 minutes, followed by 1 cycle of 98 °C for 10 minutes and cooling to 4 °C. Fluorescence of droplets were determined using the QX200 Droplet reader (Bio-Rad) and data analysed using the QuantaSoft software (Bio-Rad). Only samples with >12,000 droplets were accepted for analysis. The limit of detection was based on obtaining ≥2 droplets per primer/probe set. The amount of RNA-equivalent cDNA added to each reaction was used in the calculation of NOTCH3 +/−exon 16 transcript expressions quantified as copies/µg. The limit of detection was defined as 44 copies/µg RNA-equivalent cDNA. Since several isoforms of NOTCH3 transcripts are described^41,42^ with poor concordance of exact exon composition, expression of NOTCH3 −exon 16 was used alone or as ratios of NOTCH3 +exon 16, omitting normalizations to expression of exons positioned distant to the exact exon/region of interest.

### Statistical analysis

Prior to statistical analyses, all array based gene expression data were background corrected and normalized using the Robust Multichip Average (RMA) algorithm^43^. DLBCL patients were analysed by HG-U133 Plus 2.0 array in order to calculate subclass assignment probabilities of ABC/GCB, BAGS, and REGS using already published algorithms^7–9^. All DLBCL subtypes with n < 5 were excluded in the statistical analyses, and UC classified patients were excluded in the association analysis with ABC/GCB subclasses. The 37 patients analysed by HuEx. 1.0 ST arrays were used in identification of alternative splice variants between subclasses using the asANOVA function implemented in Partek Genomics Suite version 6.6. Lists containing genes with an alternative splice false discovery rate (FDR) corrected p < 0.05 were obtained for each classification system and the top 50 candidate genes were visually inspected. The mean gene expression of the candidate gene, NOTCH3, in DLBCL and healthy B-cells subsets were determined by extraction of hybridization-specific NOTCH3 HuEx. 1.0 probes (3853114–3853174) and subsequently, association between NOTCH3 gene expression and BAGS subtypes were investigated using unpaired t-test.

Subtype (ABC/GCB, BAGS, and REGS vincristine) specific NOTCH3 −exon 16 transcript expression patterns and association with healthy lymph node controls were analysed performing unpaired t-tests using ddPCR NOTCH3 −exon 16 expression data. To evaluate subtype specific expression variation of NOTCH3 +/−exon 16 transcripts, Person’s correlation analysis was performed for each BAGS subtype, separately. The hypothesis of vanishing Person’s correlation coefficients were tested by Fishers-r-to-z transformation. The variation was assessed by the number of data points outside the 95% confidence interval bands of the linear regression curve. The slope of the fitted regression line was used to explain the ratio of NOTCH3 +/−exon 16 transcripts. Survival analysis was performed overall and according to ABC and GCB subclasses in the R-CHOP treated cohort (n = 60) using the Kaplan-Meier method and log-rank test statistics of survival probabilities. Overall survival (OS) was defined as the date of diagnosis to the date of death from any cause. Patients were divided into two equally large groups based on a median split of NOTCH3 −exon 16 transcript expression. All statistical analyses were performed using Graphpad Prism version 7.0, where all tests were two-tailed and p < 0.05 was considered statistically significant.

## Electronic supplementary material

Supplementary Information
