# Supplementary material for: Expression of NOTCH3 exon 16 differentiates Diffuse Large B-cell Lymphoma into molecular subtypes and is associated with prognosis
Source: Sci Rep. 2019 Jan 23;9:335. doi: 10.1038/s41598-018-36680-x (PMC6344585; doi:10.1038/s41598-018-36680-x)
Supplement: Supplementary file 1 — Supplementary Information [file 41598_2018_36680_MOESM1_ESM.docx]

Supplementary Information

Expression of NOTCH3 exon 16 differentiates Diffuse Large B-cell Lymphoma into molecular subtypes and is associated with prognosis

Ditte Starberg Jespersen^1^, Anna A. Schönherz^1,4^, Hanne Due^1^, Martin Bøgsted^1,3,4^, Teis Esben Sondergaard^2^, and Karen Dybkær^1,3,4^

Supplementary figures

**
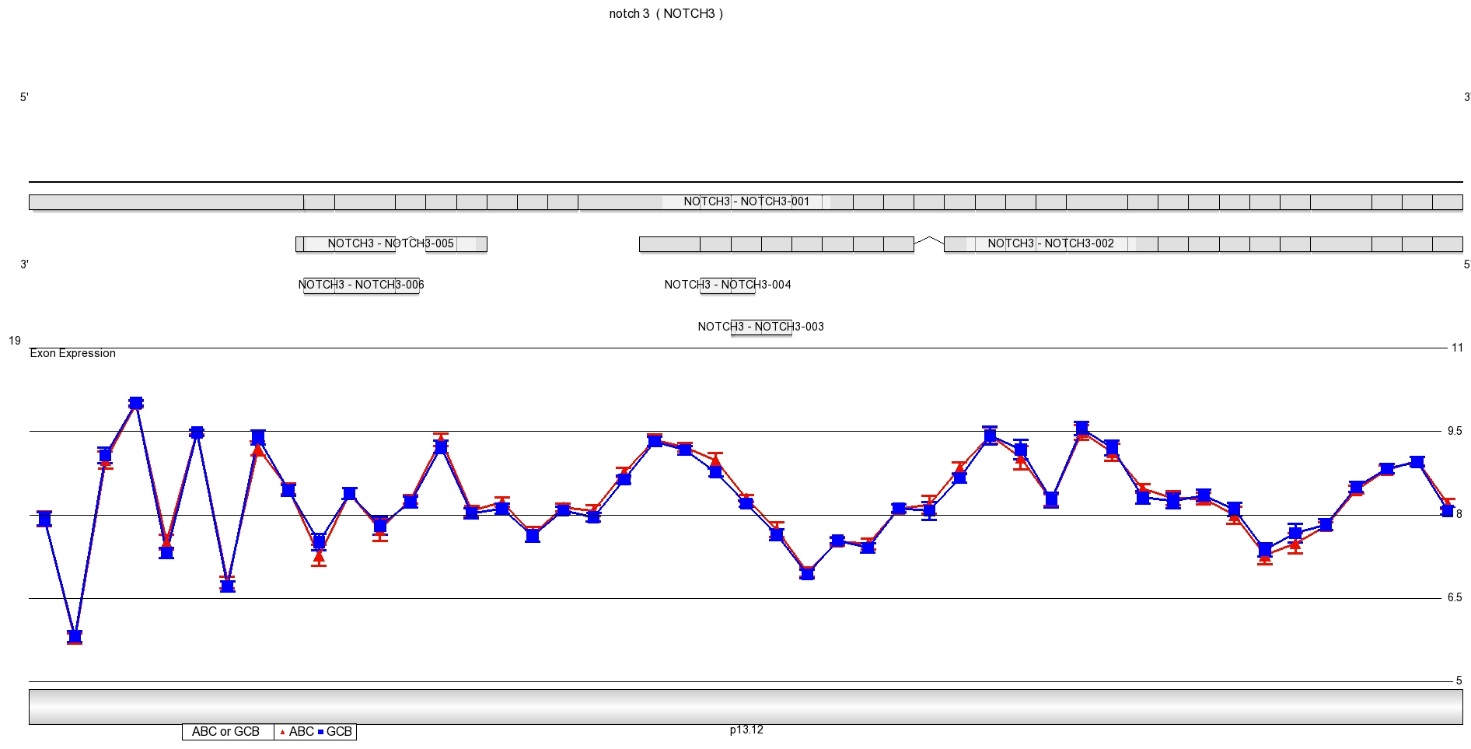
Supplementary Figure 1. NOTCH3 exon expression profiles for molecular ABC/GCB subclasses.** The upper panel shows the know NOTCH3 transcripts. The lower panel show expression profiles presenting probe-wise expression across the NOTCH3 gene for the ABC (red) and GCB (blue) subclass.

**
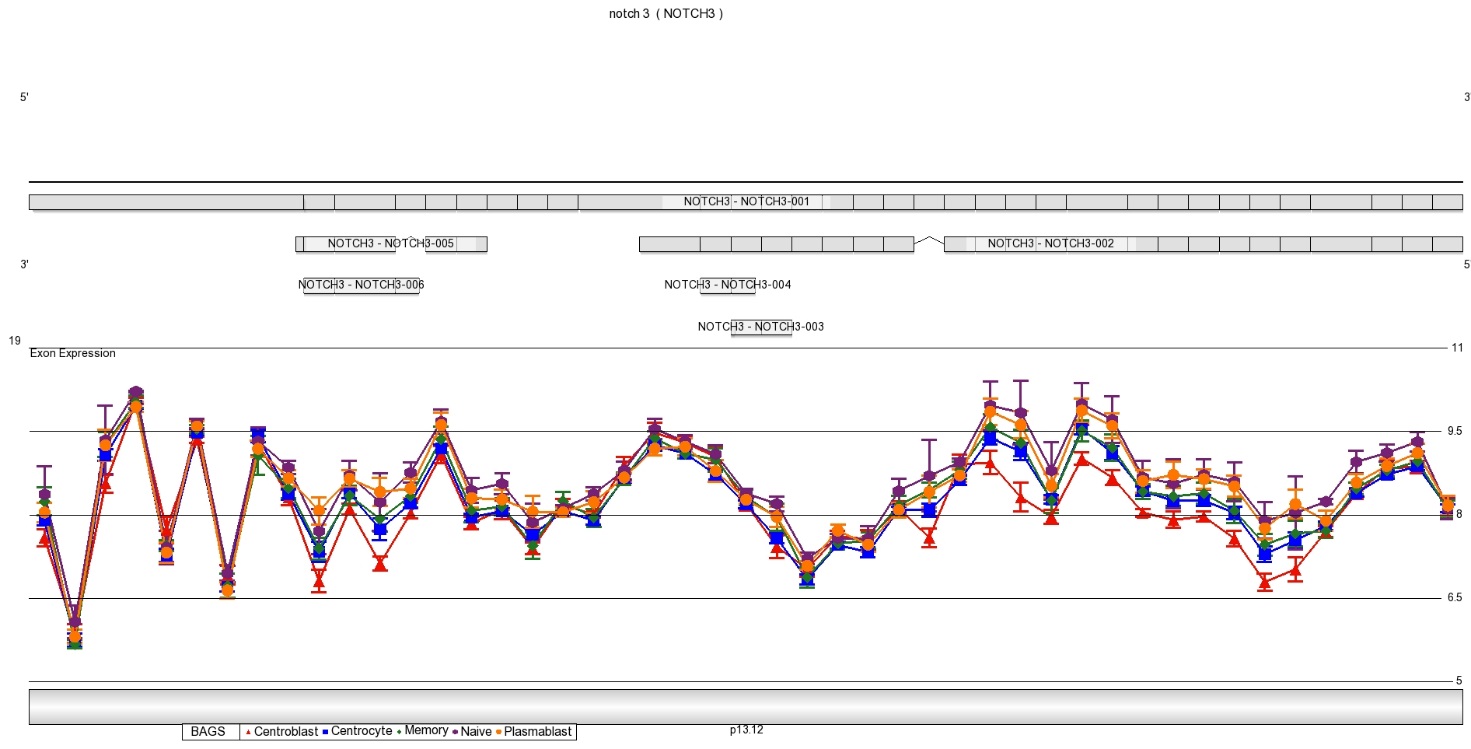
Supplementary Figure 2. NOTCH3 exon expression profiles for molecular BAGS subtypes.**

The upper panel shows the know NOTCH3 transcripts. The lower panel show expression profiles presenting probe-wise expression across the NOTCH3 gene for the BAGS subtypes: centroblast (red), centrocyte (blue), memory (green), naïve (purple), plasmablast (orange).


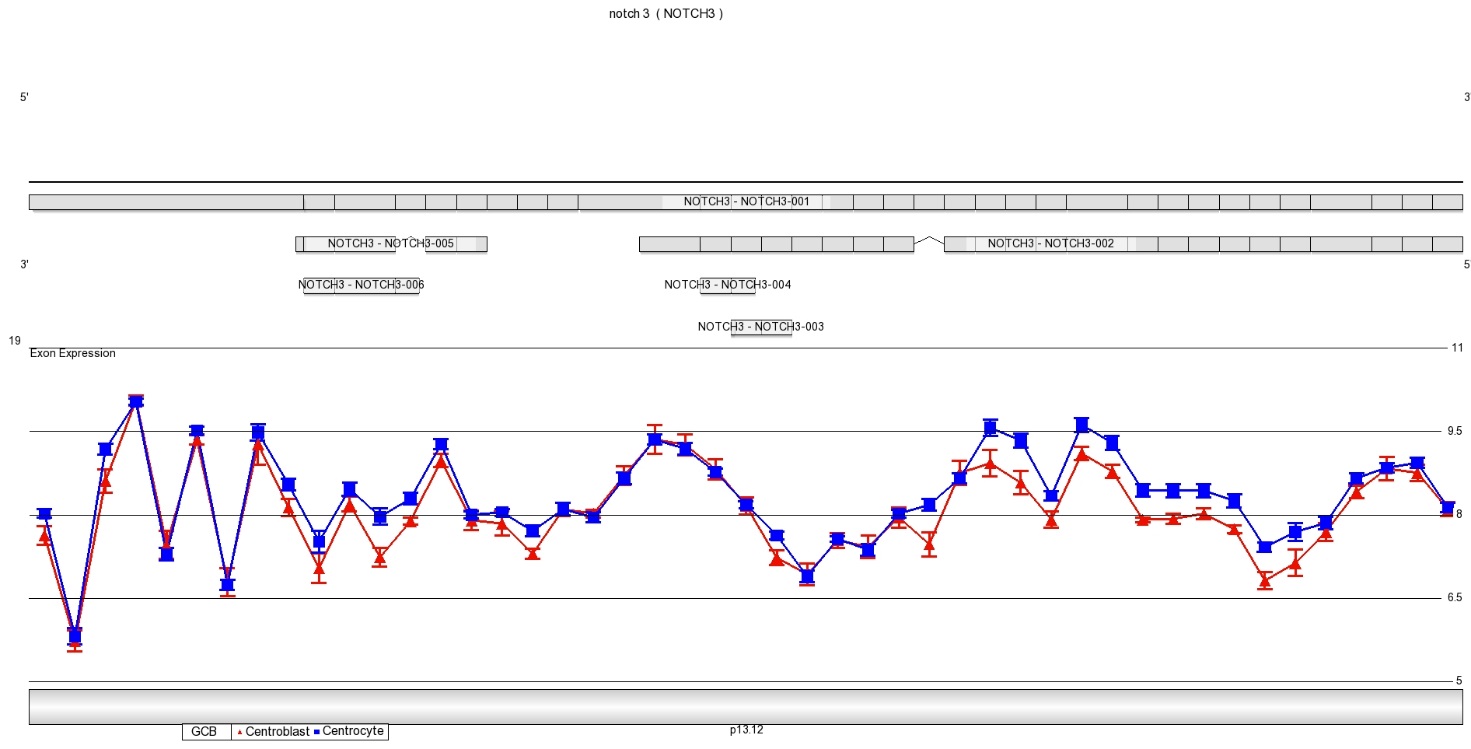
**Supplementary Figure 3. NOTCH3 exon expression profiles for GCB-centroblast and GCB-centrocyte.** The upper panel shows the know NOTCH3 transcripts. The lower panel show expression profiles presenting probe-wise expression across the NOTCH3 gene for the GCB-centroblast (red) and GCB-centrocyte (blue) subtypes.


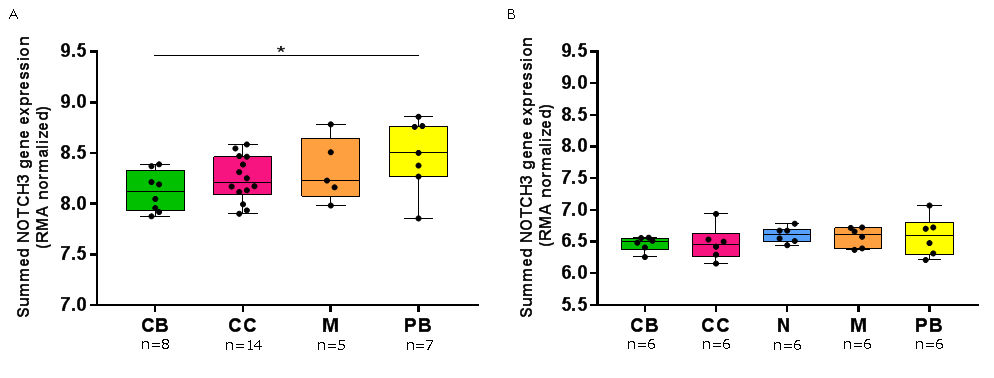


**Supplementary Figure 4. Summed NOTCH3 gene expression in BAGS subtypes from clinical DLBCL patients and healthy B-cell subsets from tonsils.** HuEx. 1.0 ST probe set expressions specific for exon 1-33 in the NOTCH3 gene (probe set ID: 3853114-3853174) were summed to a mean gene expression of NOTCH3 according to BAGS subtypes in A) clinical DLBCL patients and B) healthy B-cell subsets from tonsils. *: p-value<0.05.


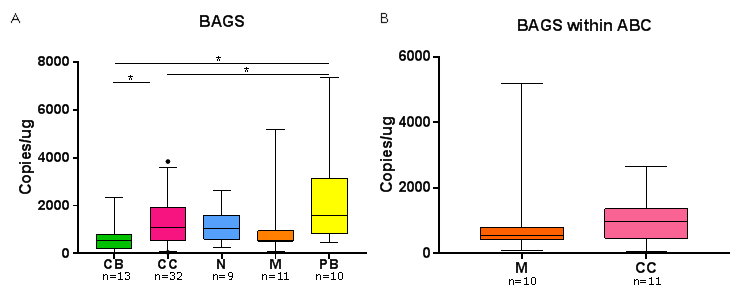


**Supplementary Figure 5. Association of NOTCH3 -exon 16 with BAGS subtypes.** NOTCH3 -exon 16 expression quantified using ddPCR associated with A) BAGS subtypes in all DLBCL patients and B) BAGS subtypes within ABC classified patients. Median Whiskers boxplots were created with 5-95 percentiles. *: p-value<0.05

Supplementary tables

| **Supplementary table 1. NOTCH3 +/- exon 16 ratio assessed as the slope of a linear regression curve.** | |
| --- | --- |
| **All** | **Slope** |
| Naive | 13.19 |
| Centroblast | 58.1 |
| Centrocyte | 25.18 |
| Memory | 25.1 |
| Plasmablast | 40 |
| **Within ABC** |  |
| Centrocyte | 31.61 |
| Memory | 24.8 |
| Plasmablast | 42.61 |
| Naïve* |  |
| Centroblast* |  |
| **Within GCB** |  |
| Centroblast | 55.89 |
| Centrocyte | 25.58 |
| Naive* |  |
| Memory* |  |
| Plasmablast* |  |
| *not possible due to low sample size. ABC: Activated B-cells, GCB: Germinal center B-cells. | |

| **Supplementary table 2. Correlation coefficient difference between BAGS subtypes and within ABC/GCB subclasses, separately.** | | |
| --- | --- | --- |
| **All** | **z-score** | **p-value** |
| Naive/Centroblast | 3.86 | 1.00e-4 |
| Naive/Centrocyte | 2.13 | 0.03 |
| Naive/Memory | 2.64 | 8.00e-3 |
| Naive/Plasmablast | 3.08 | 2.00e-3 |
| Centroblast/Centrocyte | 2.83 | 4.6e-3 |
| Centroblast/Memory | 1.20 | 0.23 |
| Centroblast/Plasmablast | 0.57 | 0.57 |
| Centrocyte/Memory | 1.18 | 0.24 |
| Centrocyte/Plasmablast | 1.80 | 0.07 |
| Memory/Plasmablast | 0.56 | 0.58 |
| **Within ABC** |  |  |
| Centrocyte/Memory | 0.20 | 0.84 |
| Centrocyte/Plasmablast | 2.13 | 0.03 |
| Memory/Plasmablast | 1.97 | 0.04 |
| Naïve* |  |  |
| Centroblast* |  |  |
| **Within GCB** |  |  |
| Centroblast/Centrocyte | 1.04 | 0.30 |
| Naive* |  |  |
| Memory* |  |  |
| Plasmablast* |  |  |
| *not possible due to low sample size. ABC: Activated B-cells, GCB: Germinal center B-cells. | | |
